# Proposed methods for testing and selecting the ERCC external RNA controls

**DOI:** 10.1186/1471-2164-6-150

**Published:** 2005-11-02

**Authors:** External RNA Controls Consortium

**Affiliations:** 1 Members of the External RNA Controls Consortium are listed in Appendix 1; 2 Send correspondance to Laura H Reid

## Abstract

The External RNA Control Consortium (ERCC) is an ad-hoc group with approximately 70 members from private, public, and academic organizations. The group is developing a set of external RNA control transcripts that can be used to assess technical performance in gene expression assays. The ERCC is now initiating the Testing Phase of the project, during which candidate external RNA controls will be evaluated in both microarray and QRT-PCR gene expression platforms. This document describes the proposed experiments and informatics process that will be followed to test and qualify individual controls. The ERCC is distributing this description of the proposed testing process in an effort to gain consensus and to encourage feedback from the scientific community. On October 4–5, 2005, the ERCC met to further review the document, clarify ambiguities, and plan next steps. A summary of this meeting and changes to the test plan are provided as an appendix to this manuscript.

## Background

### External RNA controls consortium

The External RNA Control Consortium (ERCC) is an ad-hoc group with approximately 70 members from private, public, and academic organizations. The group was initiated in 2003 to develop a set of external RNA control transcripts that can be used to assess technical performance in gene expression assays. The external RNA controls will be added after RNA isolation, but prior to cDNA synthesis. They are being designed to evaluate whether the results for a given experiment are consistent with defined performance criteria. All ERCC work is intended to apply to quantitative, real-time reverse transcriptase polymerase chain reaction (QRT-PCR) assays as well as one-color and two-color microarray experiments.

The ERCC has worked together to define the desired properties of the transcripts, general protocols for their application, and an analysis scheme for performance assessment. In December 2003, the group developed a specification document that was discussed and refined in a public workshop at the National Institute of Standards and Technology (NIST) [1]. Protocols for the use of external RNA controls in clinical applications are included in the Molecular Methods 16-P document from the Clinical and Laboratory Standards Institute, and were developed in a formal, accredited, open, consensus forum including several ERCC members. The analysis approach was developed in a public workshop at NIST in June 2004, and is based upon the measurement of pooled transcripts at known concentrations.

In the past year, the ERCC has refined specifications, generated and collected control sequences, evaluated optimal polyadenylated (polyA) tail length and identified a path forward for access and distribution of the controls. We are now initiating the Testing Phase of the project as described in this document.

### Purpose of this document

During ERCC Testing, candidate external RNA controls will be evaluated in both microarray and QRT-PCR gene expression platforms. This document describes the proposed experiments and informatics process that will be followed to authoritatively test and qualify individual controls. Qualification of a control sequence is consistent with the ISO 9000 definition of validation: "Confirmation, through the provision of objective evidence, that requirements for a specific intended use or application have been fulfilled." Based on the results of the test and qualification experiments, the ERCC will select a set of external RNA controls that perform consistently across technologies and platforms. DNA clones of the controls, basic informatics tools and appropriate documentation will be available to the public. Commercial products (e.g. primer sets and pools of transcripts) may also be developed and made available as Certified Reference Materials.

The ERCC is committed to open access and inclusive practices. We are distributing this description of the proposed testing process in an effort to gain consensus from the scientific community and to confirm the value of the final products. We hope it will be carefully reviewed by clinical and research laboratory scientists. On October 4–5, 2005, NIST hosted an ERCC Testing Workshop as an open forum to encourage feedback from the community and to invite volunteers to participate in the testing phase tasks. Comments from the meeting are posted on the NIST site [2] and included in Appendix 2 of this document. Interested parties should contact Dr. Janet Warrington [3] for further information.

### Specific aims

The five specific aims of the testing project will result in production of a well-characterized, tested set of controls with demonstrated acceptable performance on major microarray platforms and commonly used QRT-PCR methods. The specific aims are as follows.

1. Design and produce the reagents necessary for the testing plan, including candidate external RNA controls, prototype arrays, primer sets for QRT-PCR and informatics tools for managing and annotating the testing results.

2. Qualify the reagents. Identify and minimize cross reactivity and potential interactions between the external controls, the probes used for their detection and background RNA molecules.

3. Qualify the assay by collecting performance data in multiple gene expression assays. Identify the linear range, sensitivity and reproducibility of individual candidate controls. Define performance criteria and select a candidate reference set of external RNA controls for future testing.

4. Qualify the product by using the candidate reference set of external RNA controls in typical microarray and QRT-PCR assays. Extend analyses to multiple RNA backgrounds and testing sites. Confirm the performance quality of the set of transcripts and the informatics tools used for their analysis. Finalize the reference set and ERCC products.

5. Distribute DNA clones of the external RNA controls and informatics tools to general scientific community. Publish report on ERCC materials, testing data and analysis methods. Write application use and general protocols.

### Definitions, assumptions and limitations

In this document, the term "external RNA controls" refers to unlabeled, polyadenylated sense transcripts that are added to an RNA sample prior to processing and used to measure technical performance of the assay system. Although labeled transcripts will be generated during the testing effort to characterize the hybridization dynamics of each control, the final ERCC product is not intended to be used as a "hybridization control" in microarray experiments.

### Use of external controls

The external RNA controls (sometimes referred to as "spikes") are intended to be added to total RNA samples before initiating the gene expression experiment. For microarray assays, a pool of multiple external RNA controls can be added to a single sample, then labeled and hybridized in parallel with the target. If each of the added spikes is introduced at a different concentration, a calibration curve can be constructed to indicate the linear range of the hybridization. Different pools of external RNA controls can be used in separate hybridizations (one-color system) or in different channels of the same hybridization (two-color system). For QRT-PCR assays, a single external RNA control can be added to the RNA sample and amplified simultaneously with the target sequence (two-color system) or independently in a replicate well (one-color system).

#### Current external RNA controls in microarray assays

Several sets of external RNA controls are currently available for microarray research [4-9], but few probes corresponding to the controls are available on commercial microarrays and there is no consistency across platforms. As a result of the ERCC effort, a standard set of approximately 96 well-characterized, external RNA controls will be available. Many different array manufacturers are participating in the ERCC process and have committed to including probes complementary to the ERCC external RNA controls on their future microarray products.

#### Current external RNA controls in QRT-PCR assays

There are two RNA transcript quantification methods for QRT-PCR assays, absolute and relative quantification. Absolute quantification is very useful and accurate when QRT-PCR is used for measuring one or a few targets, but it often requires generation of template standards for each target and it may become cumbersome especially when many targets are analyzed [10,11].

When QRT-PCR is used for gene expression profiling, or measuring dozens of transcripts simultaneously, relative quantification is often used. In this case, users rely on internal genes for normalization, usually a "housekeeping" gene or other transcript whose expression level is thought to be invariant. This approach has its own limitations. For example, an accurate relative comparison of gene expression requires similar primer affinities for both the target and the internal control, which are often difficult to achieve. Also, recent reports [12-17] have shown that the expression levels of several housekeeping genes change in response to drug treatments or environmental changes, such as stress. These fluctuations raise questions about the general utility of a single universal set of genes as internal controls. Finally, the results of QRT-PCR reactions containing QRT-PCR inhibitors in RNA preparations are cumbersome to correct by using an internal gene transcript.

External RNA controls would provide an alternative method for normalization in relative quantification assays. The combination of external and internal or invariant controls is recommended as the most robust approach to optimal experimental control. Dose dependence, tissue specificity and degradation issues impact consistency of information and can be controlled for using a combination of controls. In addition, internal controls are more appropriately custom designed for analyte control in assays with a clearly defined intended use and therefore are by definition assay-dependent. The external RNA controls will provide a complementary resource to internal and assay-specific custom controls.

### Clone collection

The ERCC product will be a set of clones that have been well characterized for performance on multiple microarray and QRT-PCR platforms. Candidate clones are submitted to the ERCC and evaluated as described in this document. Based on the testing results, the ERCC will select clones that perform acceptably on all participating platforms to be used as the reference set.

At this time, a number of organizations have submitted 140 candidate transcripts to be tested. As shown in Table 1, the candidate external RNA clones are either synthesized from unique sequences (*i.e*., artificial) or are derived from genes in several non-mammalian species. Inserts are 500 – 2000 bp with a 20–30 bp polyadenylated tail. These clones are given freely without intellectual property rights. The ERCC welcomes additional submissions. Interested parties should contact Dr. Janet Warrington for further information [3].

**Table 1:** Summary of external RNA control clone library

***Number***	***Affiliation of Contributor***	***Genus species***	***Length of RNA***
1 – 28	Affymetrix	*B. subtilis*	700–2,000
29 – 40	Affymetrix	Artificial Sequences	500–1,900
41 – 43	USDA-ARS-NCAUR	*Bos taurus*	500
44 – 46	USDA-ARS-NCAUR	*Glycine max*	500
47	Ambion	Lamda phage	1,000
48 – 53	Ambion	Artificial Sequences	750–1,000
54 – 61	Ambion	*E. coli*	750–2,000
62 – 82	Stanford University	*Methanococcus*	500–750
83 – 85	Agilent Technologies	Artificial Sequences	500
86 – 90	GE Healthcare	*E. coli*	1,000
91 – 140	Affymetrix/Ambion/Atactic	Artificial Sequences	1,000

### ERCC products

This work culminates in the selection and distribution of materials that support the use of external RNA controls in expression assays. At the completion of the testing phase, the ERCC will release three products for use by the scientific community:

• DNA clones and sequences of the reference set of external RNA controls

• Basic informatics tools

• Publication of data, test results, protocols

Several additional reagents and documents will be developed during the testing phase. Many of the documents will be issued electronically to the general scientific community via NIST [2] as supplemental information validating the control production process. Laboratory reagents will be used exclusively by ERCC members during testing. Some of the reagents will be donated by participating ERCC institutions, including array manufacturers, reagent manufacturers and NIST.

## Methods

### Testing strategy

The testing work is divided into five sequential phases to coincide with the specific aims listed above. Milestones will be used to indicate the completion of each phase (Table 2).

**Table 2:** Summary of testing phases

***Testing Phase***	***Specific Aim***	***Milestone***
1 – Design & Development	Generate Reagents	Distribution for prototype testing
2 – Prototype Testing	Validate Reagents	Initial data collected, acceptance criteria established
3 – Proof of Concept	Validate Assay	Candidate set of ERCC clones
4 – Functional Testing	Validate Product	Final set of ERCC clones
5 – Performance Review	Distribute Product	Symposium

The proposed methods and specific tasks associated with each phase of testing are described in the following sections. The testing will be performed in parallel by three working groups: microarray group, QRT-PCR group and informatics group. A fourth group responsible for RNA production will be contracted by NIST during phase 1. All of the ERCC members will participate in the documentation and publication tasks.

### Tasks for transcript and other reagent production subgroup

This section describes the production and quality control processes for the candidate external RNA clones used in testing. NIST will be soliciting these manufacturing tasks through standard governmental procurement process.

#### Phase 1 – design and development

##### • Collect clones

DNA clones for candidate external RNA controls will be collected and stored in a NIST laboratory. Preliminary sequence information will also be collected from each clone contributor. Each clone will be assigned a unique identification number that coincides with all bioinformatic nomenclature specification requirements. A list of all candidate external RNA controls with vector, insert characteristics for the DNA plasmids and *E. coli *clones (strains carrying plasmids) will be compiled. This file will also contain the potential plasmids, which will be used for the production of the external RNA controls. A portion of the DNA plasmid or *E. coli *stock will be sent to the selected manufacturer(s) for the production of the ERCC reagents to be used in the testing project.

##### • Verify DNA sequence

Each plasmid will be grown in a small scale (~10 ml culture), purified and used for DNA sequence verification. Primer extension reactions will be designed to obtain full-length insert sequences. Both strands will be sequenced. These sequence files will be compared to the sequence submitted by the clone contributor. It will be important to verify both the presence of a polyA tail and the 3' restriction site to be used for linearization during *in vitro *transcription (IVT) template preparation. Ambiguities will be noted and will be further investigated by re-sequencing and database comparisons. The ERCC Informatics subgroup will determine the "correct" sequences to be used for microarray probe and QRT-PCR primer design. Once all of the sequences have been collated and verified, these plasmids will be used for the next phase of production.

##### • Produce bacterial stocks and plasmids

All of the clones will be grown in 1 liter cultures to generate 1–2 mg of plasmid DNA. Samples of the bacterial cultures will be saved to create two sets of triplicate glycerol stocks properly labeled and stored at -80°C. One set will be sent to NIST and one set will remain at the manufacturer's site until the completion of the project, at which time the second set will be also be returned to NIST. Each purified plasmid will undergo a set of quality control tests to measure DNA concentration, confirm purity and verify the ability to be linearized at a restriction site located 3' of the polyA tail region. This last confirmation ensures the usability of the clones in the IVT reactions. The sequence will be re-verified using a single pass primer extension from the RNA polymerase promoter sequence (*e.g*., T7, T3).

##### • Digest plasmids

Each plasmid will be linearized by restriction digestion using the appropriate amount of DNA needed to produce the specified amount of transcript. The plasmids will be inspected by high quality agarose gel electrophoresis to ensure complete digestion. The linear plasmids will be purified and concentrations will be normalized to 1 mg/ml.

##### • Test IVT template

Approximately 1 ug of each linear plasmid will be used in a 20 ul IVT reaction. The resulting RNA transcript will be diluted and inspected on an Agilent 2100 Bioanalyzer. This analysis will determine whether the size of the RNA transcript matches the expected length for each plasmid and whether any of the IVT reactions produced aborted products. At this stage, the observed transcript RNA yields will be used to calculate the efficiency of IVT reactions for each plasmid. Transcription problems will be addressed at this stage and will help dictate reaction volumes needed for the large scale IVT reactions in the next phase of production.

##### • Produce RNA transcripts

The IVT reactions will be scaled-up to produce the desired specified amount of RNA transcript, with considerations of RNA recovery and reasonable overage. Immediately after IVT, a small amount of RNA will be analyzed on the Agilent 2100 Bioanalyzer. If the transcript RNA is the expected size, it will enter into a large scale RNA purification method. The final purified RNA will be normalized to a concentration of ~1 mg/ml. These will be known as the "Production RNA Transcript Stocks".

##### • Verify quality of transcript stocks

The quality of the Production RNA Transcript Stocks will be assessed for several characteristics, including concentration, purity, integrity, stability and size. Concentration will be determined using rigorously developed standard operating procedures for UV absorbance. Purity has two aspects: the integrity of the transcript (*i.e*., percentage of full length transcript) and the stability of the transcript (*i.e*., presence of low levels of nucleases). The quality of the stock transcripts will also be confirmed by measuring the length of the RNA to determine if it matches the expected size. The Agilent 2100 Bioanalyzer will be used for analysis of integrity, stability and nucleotide length.

##### • Prepare individual stocks of RNA transcripts

The individual RNA transcripts will be diluted in RNA Storage Buffer (citrate buffer pH 6.3) and normalized to 100 ng/ul at 1000 nt in length. For example, RNA transcripts that are 750, 1200, and 2000 nt in length will be diluted to 75, 120 and 200 ng/ul respectively.

To determine the molar concentration of 1000 nt transcript, a formula or software script will be used to calculate exact molecular weight (MW) given RNA sequence. The RNA (single-stranded) molecular weight will be calculated for the phosphorylated, protonated form of the molecule using the following formula:

MW = (#A × 329.21) + (#C × 305.18) + (#G × 345.21) + (#U × 306.17) + 18.02

Stocks of individual RNA transcripts will be available in two forms: 96- well plates or screw-top tubes. Each RNA will be diluted and 50 ul aliquots of each normalized concentration will be transferred to a pre-specified well of a 96-well plate. Wells will be spot-checked for volume. Plates will be sealed with tape. Barcode tracking will be utilized to identify lots and all other tracked information. Plates will be labeled, dated and stored at -80°C until final packaging and shipping. The RNA transcripts will also be distributed in individual tubes at an equal molar concentration (to be determined).

##### • Prepare pools of RNA transcripts

A series of RNA mixtures will be made according to the experimental plans. A description of all the pools required for microarray testing is presented in Table 3. The QRT-PCR testing generally relies on a series of dilutions of equal molar pools of the RNA transcripts (pools 0 and 11 in Table 3).

**Table 3:** Description of pools and experiments in microarray testing

***Pool***	***External RNA Clones***	***Background RNA***	***No. of Arrays***
0	1 to 144	none	0
1	1 to 48 pre-labeled	none	3
2	49 to 96 pre-labeled	none	3
3	97 to 144 pre-labeled	none	3
4	1 to 144pre-labeled	none	0
5	1 to 72 (high conc.) 73 to 144 (low conc.)	human	3
6	1 to 72 (low conc.) 73 to 144 (high conc.)	human	3
7*	1 to 96 (diff. conc.)	human	12
8*	1 to 96	human	12
9*	1 to 96	human	12
10*	1 to 96	human	12
11	1 to 96	human	0
12	1 to 96	human	3
13	1 to 96	human	3
14	1 to 96	human	3
		***Total***	**75**

*Pools 7–10 may be further diluted to expand the concentration range tested.

Array count is per one-color platform and does not include background RNA negative control samples.

Pools 0 and 11 will also be used in QRT-PCR assays.

##### • Manufacture pre-labeled cRNA

The RNA transcripts will be reverse transcribed with an oligo(dT)-T7 promoter primer and second strand synthesis will be performed to create cDNA templates for the synthesis of pre-labeled cRNA for each external RNA control. To allow testing in multiple microarray platforms, three types of cRNA labels will be used: biotin, DIG and amino-allyl. This labeled cRNA will be purified and quantified as above, normalized to equal molar concentrations, and pooled as shown in Table 3 to construct accurate standard signal intensity response curves for each oligonucleotide probe feature corresponding to the external RNA controls.

### Tasks for microarray subgroup

This section describes the testing methods necessary to develop a set of external RNA controls that can be used to assess the technical performance of microarray experiments. It is anticipated that many of the microarray vendors will participate in the ERCC testing, including Applied Biosystems, Affymetrix, Agilent, GE Healthcare and Illumina. Spotted, non-commercial microarrays will also be contributed by the USDA and NCI. This collection of microarray products include both one-color platforms, which hybridize one labeled target to a single microarray, and two-color platforms, which hybridize two targets with different labels to a single microarray.

The microarray testing phases are designed to evaluate the candidate external RNA controls on both one-color and two-color platforms using the same pools of transcripts. Many of the experiments are designed to initially generate sufficient, quality data using relatively few microarrays and pools of transcripts. They may be expanded to a larger range of transcript concentrations and/or a greater number of replicate samples, as desired. A description of all required external RNA control pools is presented in Table 3. This plan assumes that phase 1 begins with 144 candidate external RNA controls and that a putative set of approximately 96 clones has been selected for further characterization by phase 3.

#### Phase 1 – design and development

##### • Design probes and generate commercial microarrays

After sequence verification, each array manufacturer will design probes targeting all of the candidate external RNA controls and generate microarrays to be used during testing.

##### • Design probes and generate non-commercial microarrays

Long DNA oligonucleotide (70-mer) probes will be designed by a joint effort of USDA and TIGR upon completion of sequence collection and *in silico *validation. The oligonucleotides will be synthesized with 5' amine modifications through a custom synthesis and spotted on glass slides.

#### Phase 2 – prototype testing

The goals of the Prototype Testing phase, addressed in two experiments, are to ensure that the RNA transcript stocks and the probes to detect each of the external RNA controls exhibit a basic level of functionality.

##### • Test probes for cross-hybridization

The first experiment is designed to check for unacceptable levels of cross-hybridization between the full set of candidate probes. To observe hybridization characteristics apart from labeling efficiencies, cRNA targets for each external RNA control will be pre-labeled with biotin, DIG or amino allyl molecules and pooled before hybridization. Assuming 144 candidate external RNA controls, the pre-labeled targets will be split into three pools (*e.g*., controls 1–48 in pool 1, controls 49–96 in pool 2 and controls 97–144 in pool 3) and a fourth pool of all pre-labeled transcripts will be generated. For one-color platforms, each pool of targets will be hybridized separately in triplicate against the test arrays. For two-color platforms, the pool 1–3 transcripts will be coupled to Cy3, while the pool 4 transcripts will be coupled to Cy5. Each test pool of Cy3-labeled transcripts will be hybridized in triplicate against reference pool 4 of Cy5-labeled transcripts.

Each pool will initially be tested in the absence of labeled background cRNA. The expected result is that probes will only show a significant signal when the target they were designed against is in the pool hybridized to the array (*i.e*., 1 of the 3 pools). Examination of the relative signals for the probes whose targets were hybridized to the arrays should allow identification of probes that show potential cross-reactivity to another sequence included in the same pool. As a negative control, three arrays will be hybridized to cRNA targets generated from at least one representative background human sample in the absence of any external RNA controls. This representative background RNA sample will be from a human tissue (or set of tissues) chosen from the Microarray Quality Control Project [18] currently underway at the FDA-NCTR. As described in the previous section, RNA from other species can also be tested for cross hybridization. Probes with minimal cross hybridization will be incorporated into the set of 96 external RNA controls that are further characterized in phase 3.

##### • Confirm labeling and dose response abilities

The second experiment in the Prototype Testing phase will test whether the external RNA controls can be labeled in the presence of a complex background of total RNA. It will also demonstrate their ability to detect known differences in transcript abundance between two pools. One pool will contain external RNA controls in one of two concentrations (*e.g*., controls 1–72 at 1:10,000 and controls 73–144 at 1:40,000) in the representative human background total RNA. A second pool will be created in which the concentrations are reversed. Both pools will go through three independent target preparation reactions and hybridizations. For one-color platforms, each labeled pool of transcripts will be hybridized to a separate microarray. For two-color platforms, targets from both pools can be labeled with different Cy dyes and hybridized to the same microarray. The observed ratios across the two pools will be compared to the expected 4-fold change. External RNA controls that do not label or give the expected response (something reasonably close to a 4:1 intensity ratio) will be removed from the pool of candidate external RNA controls.

#### Phase 3 – proof of concept

##### • Perform modified latin square and graeco-latin square experiments

During the Proof of Concept phase, specific acceptance metrics will be determined and the performance of each external RNA control will be tested over a range of concentrations. 1:5,000,000 to 1:1,000. The experimental design for this phase of array testing must meet the following three criteria: 1) Require a minimal number of arrays and pools; 2) Introduce transcripts as series of pools with balanced cRNA load that are made at a central site, rather than in individual testing labs; and 3) Use the same pools on both one-color and two-color platforms.

**Figure 1:**
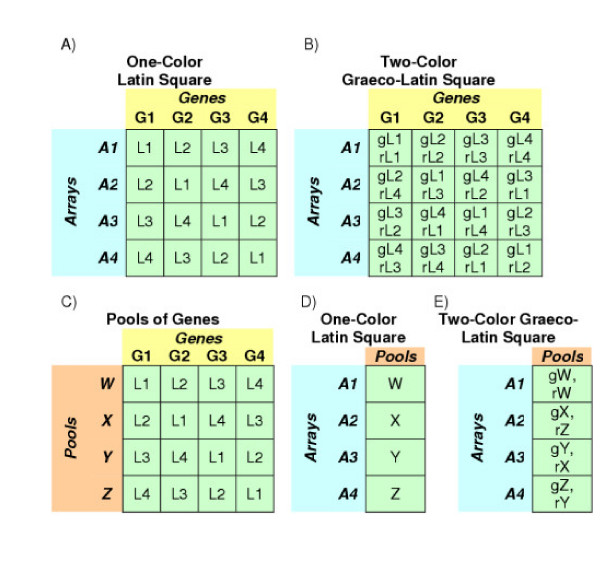
**Illustrations of latin square and graeco-latin square designs**. "A1" to "A4" number the 4 arrays used in the experiment, "G1" to "G4" number the 4 transcripts being studied and "L1" to "L4" denote 4 different concentrations for each transcript. The four pools of transcripts are labeled "W" to "Z". "g" and "r" note the gene concentrations or pools used in the green or red channel, respectively of a two-color experiment.

To best achieve these objectives, this phase of array testing is based on modified versions of a Latin Squares design for one-color platforms and a Graeco-Latin Square design for two-color platforms [19]. Illustrations of these types of experiments are given in Figure 1. Panels A and B describe a 4 × 4 experiment where four different transcripts are tested at four different concentrations. Panels C, D and E show how the same 4 × 4 experiment can be accomplished on two different platforms using the same four pools of transcripts.

Phase 3 will characterize a putative set of clones that are selected based on their performance in phase 2. In this discussion, we will assume the set includes 96 clones. These external RNA controls will be split into four groups of 24 controls each (groups A through D in Table 4). Four pools of external RNA controls will be created such that for each pool, each of the four groups of controls will be at a different concentration (*i.e*., a different relative mass). For simplicity, multiple transcripts are present at the same concentration in this modified design, rather than each transcript at a different concentration. This design can be referred to as a modified (or semi-) latin square experiment.

**Table 4:** Concentration of controls in dilution 1 pools for modified latin square experiments

***Pool***	***Concentration Group A (Controls 1–24)***	***Concentration Group B (Clones 25–48)***	***Concentration Group C (Clones 49–72)***	***Concentration Group D (Clones 73–96)***
Pool 7	125	1	5	25
Pool 8	25	125	1	5
Pool 9	5	25	125	1
Pool 10	1	5	25	125

Concentration is given as mass ratios, so that "125" represents 1:125,000 or 1 ng of RNA transcript per 125 ng of background RNA where the spike amount is adjusted for its length.

The four pools will be spiked into four independent target preparation reactions in the presence of the complex background human total RNA. A single experiment will consist of triplicate hybridizations of these four samples (see Table 5). For one-color platforms, each pool will be hybridized to a separate microarray. For two-color platforms, one pool labeled with Cy3 and another pool labeled with Cy5 will be hybridized to the same microarray. As a negative control, three arrays will be hybridized to cRNA targets generated from the background human sample without any external RNA controls. During analysis, data from both the one-color and two-color arrays will be normalized using the distribution of signals from the external RNA control probes, rather than to signals from the probes that hybridize to the background RNA. This normalization approach eliminates the need for dye swap experiments with two-color arrays.

**Table 5:** Modified latin square hybridization setup

***Controls Group***	***Pool 7***	***Pool 8***	***Pool 9***	***Pool 10***
A	Conc. 1	Conc. 2	Conc. 3	Conc. 4
B	Conc. 4	Conc. 1	Conc. 2	Conc. 3
C	Conc. 3	Conc. 4	Conc. 1	Conc. 2
D	Conc. 2	Conc. 3	Conc. 4	Conc. 1

##### • Expand the range of concentrations tested

A benefit of this simplified design is that it measures the performance of a large number of external RNA controls with only 12 arrays (plus the three negative control arrays), allowing for wider participation during this phase of testing, instead of limiting participation to those facilities that are able to run the potentially hundreds of arrays required for complete Latin Square or Graeco-Latin Square experiments. The potential drawback to this design is that it allows for measurement of only a limited number of target concentrations. For those interested in measuring the external RNA control performance across a wider range of target concentrations, the experiment can be expanded without the requirement of additional pools. This expansion can be accomplished by diluting each of the pools further to establish other concentration ranges, prior to introducing them into the background human total RNA.

Examples of three such dilution ranges are provided in Table 6. In this illustration, the four pools described in Table 3 are diluted 2-fold, 4-fold or 40-fold to generate Dilution 2, Dilution 3 and Dilution 4 pools, respectively. Sixteen possible pools for testing are generated, which are labeled based on their pool and dilution numbers: P7-D1; P7-D2; P7-D3; P7-D4; P8-D1; P8-D2; P8-D3; P8-D4; P9-D1; P9-D2; P9-D3; P9-D4; P10-D1; P10-D2; P10-D3; and P10-D4.

**Table 6:** Concentration of controls in dilution pools for expanded range experiments

***Pool***	***Conc. A Dil. 2***	***Conc. B Dil. 2***	***Conc. C Dil. 2***	***Conc. D Dil. 2***	***Conc. A Dil. 3***	***Conc. B Dil. 3***	***Conc. C Dil. 3***	***Conc. D Dil. 3***	***Conc. A Dil. 4***	***Conc. B Dil. 4***	***Conc. C Dil. 4***	***Conc. D Dil. 4***
Pool 7	250	2	10	50	500	4	20	100	5,000	40	200	1,000
Pool 8	50	250	2	10	100	500	4	20	1,000	5,000	40	200
Pool 9	10	50	250	2	20	100	500	4	200	1,000	5,000	40
Pool 10	2	10	50	250	4	20	100	500	40	200	1,000	5,000

Concentration is given as mass ratios, so that "250" represents 1:250,000 or 1 ng of RNA transcript per 250 ng of background RNA, where the spike amount is adjusted for its length.

Concentrations of the initial stock, "Dilution 1" pools are shown in Table 4.

Participants can choose to test a few or many different ranges. Testing all four ranges (*i.e*., the original stock plus Dilutions 2–4) would consume 48 arrays (plus the three negative control arrays) and would measure each of the 96 potential external RNA controls at 16 concentrations ranging from an estimated mass ratio of 1:1,000 down to 1:5,000,000.

For one-color arrays, each pool would be hybridized to a separate array, so that 48 microarrays would be required to generate triplicate sets of data. For two-color platforms, one pool labeled with Cy3 and another pool labeled with Cy5 will be hybridized to the same microarray. From these experiments we will determine the concentration range over which each target responds linearly. Additionally, we will determine the limit of detection, linear range, and resolvable fold-change across the linear range for each external RNA control. As shown in Table 7, the simultaneous hybridizations on two-color platforms enable differential expression evaluations of red/green ratios from 1/125 (0.008) to 125.

**Table 7:** Expected red:green ratios in two-color hybridizations

***Array***	***Pool in Green Channel***	***Pool in Red Channel***	***Group A Ratio***	***Group B Ratio***	***Group C Ratio***	***Group D Ratio***
1	P7-D1	P10-D1	0.008	5	5	5
2	P8-D1	P9-D1	0.2	0.2	125	0.2
3	P9-D1	P8-D1	5	5	0.008	5
4	P10-D1	P7-D1	125	0.2	0.2	0.2
5	P7-D2	P8-D2	0.2	125	0.2	0.2
6	P8-D2	P7-D2	5	0.008	5	5
7	P9-D2	P10-D2	0.2	0.2	0.2	125
8	P10-D2	P9-D2	5	5	5	0.008
9	P7-D3	P9-D3	0.04	25	25	0.04
10	P8-D3	P10-D3	0.04	0.04	25	25
11	P9-D3	P7-D3	25	0.04	0.04	25
12	P10-D3	P8-D3	25	25	0.04	0.04
13*	P7-D4	P7-D4	1	1	1	1
14*	P8-D4	P8-D4	1	1	1	1
15*	P9-D4	P9-D4	1	1	1	1
16*	P10-D4	P10-D4	1	1	1	1

*Self-to-self hybridizations

Pools in each channel are labeled based on their pool and dilution numbers in Table 6.

Groups are sets of transcripts at the same concentration as defined in Table 4.

#### Phase 4 – functional testing

##### • Test dose response curve pools

A typical dose response curve (DRC) experiment consists of testing each external RNA control at multiple concentrations, one per array, requiring several arrays. In contrast, a single array DRC experiment consists of multiple concentrations and multiple external RNA controls per concentration, all tested on a single array. Because the entire experiment is contained within a single array, each external RNA control is measured only at a single concentration. Therefore, the intensities from all of the probes are used together to construct the DRC across the entire concentration range. During the Functional Testing phase, the target metrics determined during the Proof of Concept phase will be used to create a single-array DRC external RNA control pool. An example single array DRC pool is shown in Table 8.

**Table 8:** Example single array DRC pool

***Concentration***	***No. of Targets***
1,000	2
2,000	2
4,000	2
5,000	0
10,000	4
20,000	4
25,000	0
40,000	10
50,000	0
100,000	12
125,000	0
200,000	12
250,000	12
500,000	12
1,000,000	12
5,000,000	12
***Total***	**96**

Concentration is given as mass ratios, so that "1,000" represents 1:1,000 or 1 ng of RNA transcript per 1,000 ng of background RNA where the spike amount is adjusted for its length

To keep the ratio of external RNA controls to background RNA as low as possible, the higher concentrations of external RNA controls (*e.g*., 1:1,000) are under-represented compared with the lower concentrations (*e.g*., 1:100,000). Additionally, external RNA controls originating from the same species will be evenly distributed across the concentration range to minimize the effects of removing them from experiments performed with background RNA from the same species.

Up to three pooling schemes will be tested, each with triplicate hybridizations. For one-color platforms, each labeled DRC pool of transcripts will be hybridized to a separate microarray. For two-color platforms, targets from different DRC pools can be labeled with different Cy dyes and hybridized to the same microarray (*e.g*., pool 12 and 13 or pool 13 and 14).

##### • Perform forced failure experiments

Once the final single array DRC pool has been chosen, a small series of forced failure experiments will be carried out to mimic how the system will respond to common failure modes (*e.g*., incorrect temperature, incorrect buffer composition, extreme washing stringency, *etc*.).

#### Phase 5 – performance review

##### • Review and publish the experimental results

Results will be shared within the ERCC for review and evaluation to determine if additional data or redesign is indicated.

##### • Repeat experiments in different labs

### Tasks for QRT-PCR subgroup

The QRT-PCR testing phases are designed to use the same pools of transcripts developed for microarray testing and to evaluate the candidate external RNA controls on multiple QRT-PCR platforms. There are two commonly used methods of detecting the QRT-PCR amplicons: DNA-binding dyes (*e.g*., SYBR Green I) and target-specific probes with fluorescent 5' exonuclease activity (*e.g*., TaqMan^®^). The testing plan will generate data on both platforms, using the same primer sets. The DNA-binding detection assays will use a single tube, one enzyme (rTth) and SYBR Green I based protocols. The target-specific detection assays will use a two-step protocol developed for the TaqMan^® ^system.

#### Phase 1 – design and development

##### • Design QRT-PCR primers and detection probes

All primers for external RNA controls will be designed using commonly used software. Two sets of QRT-PCR primers (one at 3' and one at 5' end) will be finalized and used to determine the entirety and specificity of RNA transcripts. For target-specific detection assays, a fluorescent 5' exonuclease detection probe will also be designed. The sequences of all QRT-PCR reagents and their locations on the RNA transcripts as well as the sizes of amplicons and their predicted Tm will be provided to the community. All primers and detection probes will be blasted against Genbank for potential cross-reactivity.

##### • Define cycling parameters

With the DNA-binding detection assays, the concentration and ratio of primers must be optimized to prevent fluorescent signal from "primer dimers" or nonspecific amplicons. Experiments will be performed to develop amplification parameters for ABI 7900HT and Stratagene MX3000 using a single tube, one enzyme (rTth) and SYBR Green I based QRT-PCR protocol. With the target-specific detection assays, the primer and detection probe concentrations and thermal cycling conditions will be as suggested by the manufacturer. cDNA will be generated using standard kits and random primers prior to amplification.

##### • Assist in verifying quality of transcript stocks

QRT-PCR will be used to assess the quality of the RNA transcripts produced for each of the candidate external RNA controls. Individual transcripts will be evaluated for entirety, stability, and DNA contamination. The specificity of each primer set and detection probe will be determined by amplifying individual transcripts in equal molar pools of the candidate controls. QRT-PCR can also be used to verify transcript ratios in pools with variable concentrations of the candidate controls.

#### Phase 2 – prototype testing

##### • Test primers for cross-reactivity

Each set of QRT-PCR primers as well as any detection probes will be tested for cross-reactivity with other external RNA transcripts by amplifying its perspective RNA transcript from three different RNA sources. First, individual transcripts will be amplified from a pool containing multiple RNA transcripts at equal molar concentration (pool 0 in Table 1). Second, individual transcripts will be amplified from the same pools of multiple RNA transcripts at equal molar concentration introduced into a human total RNA background. The human background RNA increases the complexity of the reaction and may be based on a reference RNA sample, for example a pool of RNA derived from 10 different human tissues. Third, individual transcripts will be amplified from the human total RNA that lacks external RNA controls. All amplicons will be examined by gel electrophoresis or melting dissociation curves. The Ct values of amplification for each transcript under other RNA background should be very similar to those with only pure RNA transcripts. If any QRT-PCR reagent gives unexpected products, the primers and/or the detection probe will be re-designed and re-tested.

##### • Optimize efficiency of QRT-PCR

A 10-fold serial dilution from 10^8 ^copies to 1 copy will be made for a pool of all candidate RNA transcripts in an equal molar concentration. The RNA dilutions will be used for examining the amplification efficiency of at least one QRT-PCR primer set, and for some platforms the corresponding detection probe, for each RNA transcript. Ideally, with a given dilution of the pool, all QRT-PCR primer sets will amplify their perspective targets with similar efficiency. Otherwise, the primer sets and detection probes with a low efficiency should be re-designed and re-tested.

##### • Determine limit of detection

Since all molecular weights and sequences are known, the physical copies of each RNA transcript in the pool of all candidate RNA transcripts can be calculated. One RNA transcript will be chosen as a standard for concentration determination and the relative concentrations of other RNA transcripts will be determined based on this standard. All possible efforts should be made to determine the copy number of each transcript by QRT-PCR close to its physical copy number of the RNA transcript in the pool. The lowest concentration of each transcript in the pool of all candidate RNA transcripts, which can be detected by QRT-PCR amplification, can be considered as the limit of detection for that transcript.

##### • Assist in verifying quality of pools

QRT-PCR will be used to assess the ratios of different RNA transcripts in different pools. Note that due to different amplification efficiencies in RT and PCR, there may be a discrepancy between the ratios determined by QRT-PCR and those measured by physical quantity.

#### Phase 3 – proof of concept

##### • Establish acceptance criteria for RNA transcripts

Based on the phase 2 results, a set of up to 96 external RNA controls will be selected for further testing. Using the 10-fold serial dilutions of a pool of these 96 RNA transcripts (pool 11 in Table 1), limit of quantification, linearity, precision and accuracy for each of RNA transcript will be determined, and the acceptance criteria of assay performance for each RNA transcript will be established. Those criteria should be established for the pure RNA transcripts as well as for the pure RNA transcripts under a background of other total RNA, such as human RNA reference.

#### Phase 4 – functional testing

##### • Compare QRT-PCR platforms and instruments

The QRT-PCR testing will generate data from both DNA-binding and target-specific detection assays on multiple thermal cycling instruments, including ABI 7000, ABI 7900HT and Stratagene MX3000. The results will be useful to evaluate the technical performance between those different instruments and platforms.

##### • Compare microarray and QRT-PCR platforms

Assay performance of a set of selected external RNA transcripts or pools with the same or different concentrations will be evaluated by microarray and QRT-PCR. The results will be very useful to guide the comparison of experimental results generated by microarray and QRT-PCR.

##### • Optimize external RNA concentrations

External RNA controls can be used for monitoring the amplification processes or for detecting the presence of possible inhibitors in QRT-PCR amplification. Optimal concentrations of external RNA transcripts should be established so that the concentrations of the introduced external RNA controls are low enough to detect the presence of possible inhibitors, but not too low that the variation of the assay would be indistinguishable from the low assay performance caused by inhibitors. Different concentrations of a given external RNA transcript will be spiked in total human RNA. The optimal concentration of an external RNA transcript would give a Ct about 30–32. Clinical RNA specimens extracted from different tissues and purified by different sample preparation methods can be used for evaluating the optimal concentrations of external RNA transcripts.

##### • Conduct forced failure experiments

To establish the utility of external RNA controls for detecting potential QRT-PCR inhibitors, a few known QRT-PCR inhibitors, such as ethanol and guanidine thiocyanate, will be introduced into QRT-PCR reactions along with external RNA controls. An impaired QRT-PCR reaction of external RNA controls could indicate the presence of possible QRT-PCR inhibitors in the RNA preparation.

##### • Evaluate multiplex QRT-PCR assays

Multiplex assays are possible when different reporter dyes are coupled to different target-specific probes so that cleavage of the multiple probes can be detected in a single PCR. These multi-color assays may also be performed.

#### Phase 5 – performance review

##### • Review and publish the experimental results

##### • Repeat experiments in different labs

To establish the reproducibility of the research and diagnostic utilities of external RNA controls, the experiments described in phase 4, multi-site testing will be performed.

### Tasks for informatics subgroup

The ERCC is committed to delivering an informatics approach that can be used to establish the technical performance of an expression measurement through the analysis of the measured response of external spiked-in RNA controls. This approach will be implemented and delivered in an open-source manner, such as R code for a bioconductor package.

The informatics requirements for ERCC development will include sequence bioinformatics links to public annotation databases. Sequence informatics will be delivered via these public resources.

The scope of ERCC informatics activity will not include examination of the application of external RNA controls for purposes other than evaluation of the technical performance of an expression measurement. Such applications as normalization to spike-in controls, or calibration, or evaluation of selectivity and specificity, while potentially useful, are left to assay development efforts for specific intended use applications.

#### Phase 1 – design and development

##### • Develop sequence bioinformatics

The sequence data will need to be managed in a sustainable fashion using established tools and approaches. At this stage, sequence bioinformatics includes both a nomenclature system and a maintainable archive of sequences. The nomenclature system requires unique identifiers as well as consistent annotation for RNA control sequences and the related PCR reagents and microarray probes across various platforms. A preliminary nomenclature system is presented in Table 9.

**Table 9:** Illustration of a nomenclature system

***Reagent***	***Nomenclature***	***Legend***
RNA Transcript	ERCC-nnnnn-vv	nnnnn = unique 5-digit sequence number vv = 2 digit version number
PCR primer/probe microarray probe	ERCC-nnnnn-vv- pppp-lll-aaa	pppp = 4-digit positional location relative to the 0^th ^base at the 5' end of the transcript sequence lll = primer/probe length aaa = nucleic acid sequence of the initial triplet of the primer/probe (complement of the RNA sequence at pppp, pppp+1, pppp+2)

The sequence archive requirements are as follows: 1) data will be stored in FASTA format; 2) where appropriate, annotation will include unambiguous cross-references to gene identifiers in public databases; and 3) maintainability and version control.

##### • Explore analytical approaches

The Analytical Informatics work in this phase is exploratory and will establish prototype tools for the potential analysis approaches. The major tasks for the analytical informatics work at this phase are: 1) identify appropriate QRT-PCR analysis approaches; 2) investigate various analysis approaches for microarray technical performance, including evaluation of preprocessing strategies (*e.g*., data normalization); 3) develop prototype tools that can be used for the exploration of the analysis approaches; and 4) test prototype tools using existing or synthetic data.

**Figure 2:**
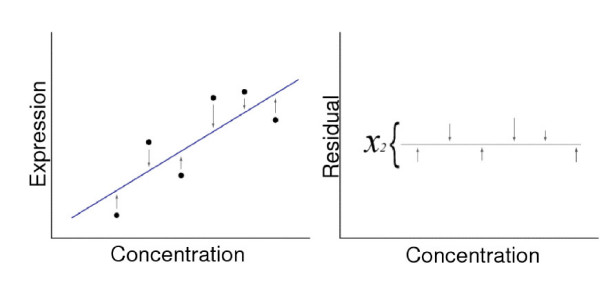
**Illustration of chi square fit**. Panel A. The distances from a straight-line fit (arrows) are calculated. Panel B. The Chi square fit of the distances is then determined.

The analysis approaches that will be explored in this phase will include:

Approach 1 – chi square fit of a linear portion of a calibration curve from the external RNA controls (Figure 2).

**Figure 3:**
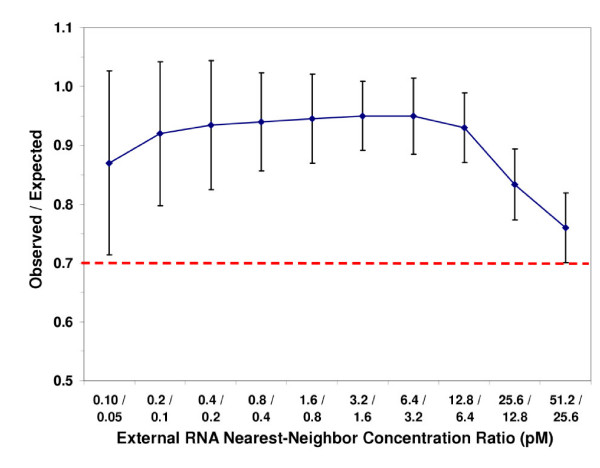
Illustration of spike performance.

Approach 2 – ratio of observed/known concentration ratios between nearest-neighbor spikes versus concentration of low spike in pair (Figure 3).

Approach 3 – linear range

1. To characterize an external RNA transcript response we can describe signal in response to target concentration by the following general function.



Here, *S *– signal from microarray element, *a *– variable incorporating parameters such as number of probes per microarray element, number of fluorescent label molecules per target molecule, power of light etc., *φ *– a function describing thermodynamics of hybridization isotherm and this function has a vector of parameters () which is unique for each probe, *c *– concentrations of spike-in target, *b*-background due to dark detector counts and sources of light other than labeled targets.

2. Let *μ*_*s*_*(c) *be a function describing relationship between expected signal and spike concentration. Also, let *ε*_*S*_*(c) *be a function describing relationship between standard deviation of signal and spike concentration.

3 Define signals S and S* (S < S*) as reliably resolved if



Define concentration levels  and  ( <) as reliably resolved if expected signals produced by these concentrations are reliably resolved.

Define concentration fold change –  as reliably resolved if concentration levels  and  are reliably resolved.

Define linear range as  where  and  are the ends of the longest interval of target concentrations such that *f *(*c*_*t*_) ≤ 2 ∀ *c*_*t *_∈ (, ).

Thus, the linear range is the largest continuous interval of target concentrations such that a two fold increase of concentration anywhere within this interval gives rise to reliably resolved expected signal levels.

4. Using these assumptions we will explore several models to determine linear range and minimal reliable resolved concentration fold change.

**Figure 4:**
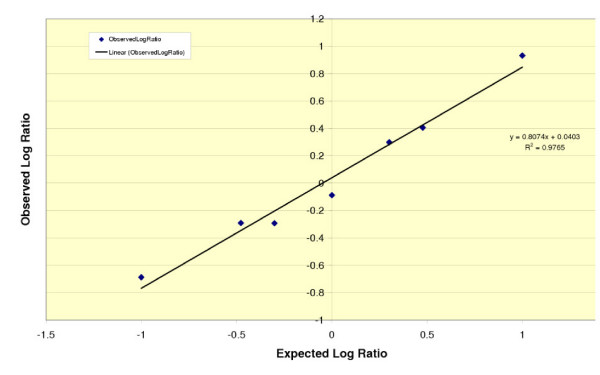
**Illustration of model data (including modeled noise)**. The values of m and b that were input into the model were m = 0.85 and b = 0.08. The noise model is realistic, in that it includes both constant (scanner) and proportional (chemical) noise.

Approach 4 – fitting the spike-in probe behavior on an individual array to a reference model of known, acceptable performance (Figure 4).

For 1-color arrays signal intensity can be described as:

Log_2_*I *= target concentration + probe affinity + background + *E*

A reference dataset can be used to estimate probe affinity and background for each of the probes or probe sets. This should allow for a more accurate estimation of the target concentration of the external RNA controls.

For 2-color arrays we can use the following model to analyze spike-in ratios:



Where *m *and *b *are the slope and intercept from a least-squares fit.

This model has the nice feature of physically reasonable and appealing interpretations of the slope and intercept. The intercept is the log of the bulk normalization constant for the external RNA control (*i.e*., the model is self-normalizing, independent of the normalization of the background samples carrying the spike-ins). The slope measures ratio flattening (*i.e*., reduction of the absolute value of the log ratio from its expected value, due to such effects as cross-hybridization of other targets to the spike-in probes). The model clearly differentiates between failures to observe expected ratios that are due to normalization and effects that are due to ratio flattening. This is important, because the two effects are often confused and misdiagnosed.

Approach 5 – ANOVA modeling.

There are several potential approaches to analyzing the variance in the experiment:

• The variability between repeated probes on the same array. This will give an indication of the variability due to spatial distribution and spot deposition.

• Other ANOVA models are possible depending on the experimental design and the variables captured in the experiment.

Approach 6 – additional robust measures for performance metrics will be explored.

#### Phase 2 – prototype testing

##### • Develop and test prototype analytic code

Implementations for the analytical approaches will be developed in the prototyping phase. These implementations will be tested against modeled/simulated data, as well as against existing data, where possible. As data become available from the QRT-PCR and microarray testing activities, those data will be used to evaluate the prototype analytical implementations. The analyses may be implemented with a variety of numeric and graphical tools (*e.g*., spreadsheets or proprietary statistical tools).

#### Phase 3 – proof of concept

##### • Qualify analytic models

At this stage of the project, data will become available from the array and QRT-PCR testing activities. These data will be used to test the prototype implementations of the analysis approaches, and those approaches will be refined as appropriate. Refinements will be tested and qualified. At the end of this stage, the approaches will be sufficiently refined to move forward to implementation for functional testing.

#### Phase 4 – functional testing

##### • Prototype open source code for analytics

This stage of the project will focus on developing implementations of the analysis approaches in R as a bioconductor package. This package should be validated to work with array data from the variety of microarray platforms being tested. Refinement of the graphical display of analysis results will emphasize platform-to-platform consistency where possible (so similar analytical graphs are presented for 1- and 2-color, and the variety of platforms). The QRT-PCR analysis approach may be developed as a separate bioconductor package, with the emphasis likely to be on a graphical display of performance measures as a time series.

##### • Develop web-based interface for analytics

Development of web-based implementations of the analysis implementations will be investigated, and common data formats for input and output will be specified and implemented.

##### • Qualify final analytic strategy

#### Phase 5 – performance review

##### • Publish bioconductor package

The bioconductor package will be published and released to the community. A manuscript will accompany publication of the package, describing the analytical approaches embodied in the package, and demonstrating performance with the validation data. All relevant performance data from the testing and development of the ERCC analytical informatics will be collated and published at this time.

##### • Publish sequence database

The reference sequence database will be published and made available on the web in both flat-file formats and a common sequence database format (FASTA).

##### • Publish relevant performance data

Performance data from the testing will be published. Collate performance data (from Test Reports), including probe performance data, in backgrounds.

### Test plan tasks for ERCC

• **Identify least burdensome path for collection and distribution of data**

• **Organize analysis jamboree**

• **Plan publication of results and timing for publication(s)**

• **Organize controls symposium**

## Conclusion

The tasks described in this document have been designed and reviewed by many ERCC members. They represent our best consensus at this time, but may not be the final format of testing. A summary of the issues discussed at the ERCC Testing Workshop on October 4–5, 2005 is provided in Appendix 2 and copies of the presentations are posted on the NIST website [2]. Additional comments are welcome and should be sent to Dr. Janet Warrington [3].

## Abbreviations

ERCC, External RNA Control Consortium

QRT-PCR, quantitative, real-time reverse transcriptase polymerase chain reaction

NIST, National Institute of Standards and Technology

polyA, polyadenylated

IVT, *in vitro *transcription

MW, molecular weight

DRC, dose response curve

Cy, Cyanine Dye

Ct, Cycle Threshold

## Appendix

### Appendix 1: Members of the external RNA controls consortium (Table 10)

**Table10:** Members of the external RNA controls cosortium

Anne Bergstrom Lucas	Agilent Technologies, Inc.
Anne R. Kopf-Sill	NuGEN Technologies, Inc
Bin Chen	Centers for Disease Control and Prevention
Bud Bromley	ViaLogy Corp.
Carole Foy	LGC Ltd
Cecelia S. Hinkel	Centers for Medicare Medicaid Services
Cecilie Boysen	ViaLogy Corp.
Chunmei Liu	Affymetrix Inc.
Daya Ranamukha-arachchi	FDA/CDRH/OSEL Division of Biology
Elizabeth Wagar	UCLA
Ernest S. Kawasaki	NCI/NIH
Federico M. Goodsaid	CDER/FDA
Friederike Wilmer	QIAGEN GmbH
Gavin Fischer	Stratagene
Gretchen L. Kiser	GE Healthcare
Helen C. Causton	Clinical Sciences Centre/Imperial College Microarray Centre
James C. Fuscoe	NCTR/FDA
James D. Brenton	University of Cambridge
Janet A. Warrington	Affymetrix, Inc.
Jesus Soriano	ATCC
John Coller	Stanford University
John D. Burrill	Applied Biosystems
Kate Rhodes	Cyntellect Incorporated
Kathleen F. Kerr	University of Washington
Kathryn C. Zoon	NIAID/NIH
Kathy Lee	Applied Biosystems
Laura H. Reid	Expression Analysis, Inc.
Leming Shi	NCTR/FDA
Marc Salit	NIST
Mary Satterfield	NIST
Matthew Marton	Rosetta Inpharmatics, LLC
Maureen Cronin	Genomic Health, Inc.
Michael P. Conley	Enzo Life Sciences, Inc.
Mickey Williams	Roche
Mike Fero	Stanford University
Mike Wilson	Ambion, Inc.
Natalia Novoradovskaya	Stratagene
Patrick Gilles	Invitrogen
Paul K. Wolber	Agilent Technologies, Inc.
Pranvera Ikonomi	American Type Culture Collection
Raj Puri	FDA/Center for Biologics Evaluation and Research
Richard P. Beyer	University of Washington
Richard Shippy	GE Healthcare
Robert Setterquist	Ambion, Inc.
Rosalie K. Elespuru	FDA/CDRH/OSEL Division of Biology
Shawn C. Baker	Illumina, Inc.
Stephen A. Chervitz	Affymetrix, Inc.
Steven R. Bauer	FDA/Center for Biologics Evaluation and Research
Steven Russell	University of Cambridge
Tamma Kaysser-Kranich	GE Healthcare
Theo K. Bammler	University of Washington
Thomas B. Ryder	Affymetrix, Inc.
Timothy J. Sendera	GE Healthcare
Uwe Scherf	CDRH/FDA
Xiaolian Gao	Atactic Technologies
Xiaoning Wu	Roche Molecular Systems, Inc.
Xu Guo	Affymetrix, Inc.
Z. Lewis Liu	USDA-ARS-NCAUR

### Appendix 2: Summary of ERCC test plan workshop

The ERCC reviewed feedback on the Proposed Testing Plan at a NIST-hosted workshop on October 4–5, 2005. The meetings were attended by more than 50 participants, including ERCC members from Europe (Belgium, Germany, UK) and the US. During the first day of the meeting, summaries of the test plan were presented by representatives from each of the four subgroups. Suggested improvements and possible testing issues were discussed as listed below:

#### Reagent production

Perhaps the ERCC set should include different types of controls (less than 100 bp or no polyA tail) that would support new labeling technologies or gene expression applications. Many members liked this possibility, and although it is outside the scope of the current ERCC initiative, it may develop into future ERCC activities. A request was made for early release of the submitted but not confirmed sequence of the external RNA controls. Another ERCC deliverable describing how to validate the quality of the external RNA controls was suggested.

#### Microarrays

The external RNA control concentrations used during phase 2 cross hybridization experiments as well as the exact definition of cross-reactivity were discussed. It was noted that identifying the specific transcript responsible for cross hybridization observed in a pool of 24 candidates may require many subsequent hybridizations with individual controls. Early definitions of the concentrations in the Latin square and dose response curve experiments are needed, with an emphasis on accommodating the ranges of both channels in two-color arrays. After a thorough discussion, it was decided to include pre-labeled external RNA controls in phase 2 of testing. Many of the microarray technology developers indicated that the test arrays might include multiple probes or probe sets in order to identify the best performing sequences. This format was acceptable, as long as participants agreed not to change probe sequence between phases.

#### QRT-PCR

The proposed test plan uses dilutions of equimolar pools of 96–140 the candidate RNA controls in the QRT-PCR assays. At the meeting, it was decided that plates of individual RNA transcripts would be more useful during the initial testing in phases 1–4. It was recognized that only 1–3 external RNA controls at a time would be used in QRT-PCR assays due to limitations in the number of fluorophors that can be detected. There was also some discussion that QRT-PCR assay optimization may require more than 2 primer sets described in the proposed test plan. A new application for the controls in the validation of thermal cyclers and other QRT-PCR instruments was discussed.

#### Informatics

The ERCC will need a plan for data storage and distribution. NCI, NCBI, and FDA representatives volunteered to provide a data repository for Test Plan data. A working group will be formed to identify needs of the ERCC and identify which of these data repositories would be a good fit. Participants were directed to the CLSI MM16-P document for a list of possible performance metrics. Preliminary review of these metrics could be initiated now using already available gene expression data on many of the candidate external RNA controls. There was some discussion on whether the external RNA controls should be optimized and selected to have the best possible results (e.g. perfect dose response curve, no cross hybridization) or left imperfect to better reflect typical probes or probe sets on the microarrays.

After nine hours of review and discussion, consensus was achieved on the ERCC Test Plan by a show of hands with no major outstanding issues. On the second day of the meeting, preliminary resource requirements were identified and the scope of testing (sample number etc.) was better defined. Key features of this discussion are summarized below:

1. In an effort to efficiently utilize the RNA reagents, early stages of testing (Phases 1–4) will be performed only at developer sites and limited to one site per platform. We estimate that up to 10 microarray and 4 QRT-PCR commercial developers will participate. If resources permit, 3 or 4 noncommercial development sites (e.g. NCI or USDA) will be included to represent home-made, spotted microarrays.

2. Phase 5 will be divided into two parts with separate goals. In Phase 5A, the last experiment of Phase 4 will be repeated at user sites to confirm that results can be reproduced using the same materials and protocols as at the developer sites. In Phase 5B, a set of the best performing external RNA controls will be distributed to user sites and incorporated into typical experiments with their materials and protocols. The intent is to establish performance of the external RNA controls in a broad variety of routine applications and protocols. One possible Phase 5B experiment would be to repeat a previous gene expression experiment to validate that similar differential expression results are observed with and without the inclusion of external RNA controls.

3. A tentative list of criteria for technology developers and user test sites was developed. It was decided that all sites should have the following attributes: 1) Demonstrated experience in gene expression platform; 2) Ability to contribute materials and labor; 3) Commitment to deposit all testing data in publicly available database; and 4) Agreement to meet ERCC timeline. In addition, developer sites should have demonstrated capacity to design and create the necessary reagents as well as a commitment to follow the test plan and collect data on all candidate clones. User test sites may be asked to contribute a novel application to the ERCC goals and to provide sample material for the complex RNA background. It is not necessary to have contributed sequences to be considered as a test site.

4. While the external RNA controls are likely to be useful in RNA samples from a variety of species, testing will emphasize human, mouse and rat RNA. A Stratagene representative offered to provide universal reference RNA samples from these three species to be used as the complex RNA background in Phases 1–4. Test microarrays will include probes or probe sets for both the candidate external RNA controls as well as genes included on their typical human, mouse and rat arrays (as determined by each developer). This design will enable global normalization methods and aid in cross hybridization experiments. The developers may elect to pre-screen their test arrays using RNA from a variety of species.

Action items and next steps will be further discussed in the monthly ERCC conference calls.

## Note

^3^Certain commercial equipment, instruments, or materials are identified in this document. Such identification does not imply recommendation or endorsement by the National Institute of Standards and Technology, nor does it imply that the products identified are necessarily the best available for the purpose.
